# Delineating Genetic Alterations for Tumor Progression in the MCF10A Series of Breast Cancer Cell Lines

**DOI:** 10.1371/journal.pone.0009201

**Published:** 2010-02-15

**Authors:** Mitsutaka Kadota, Howard H. Yang, Bianca Gomez, Misako Sato, Robert J. Clifford, Daoud Meerzaman, Barbara K. Dunn, Lalage M. Wakefield, Maxwell P. Lee

**Affiliations:** 1 Laboratory of Population Genetics, Center for Cancer Research, National Cancer Institute, Bethesda, Maryland, United States of America; 2 Laboratory of Cancer Biology and Genetics, Center for Cancer Research, National Cancer Institute, Bethesda, Maryland, United States of America; 3 Basic Prevention Science Research Group, Division of Cancer Prevention, National Cancer Institute, Bethesda, Maryland, United States of America; Innsbruck Medical University, Austria

## Abstract

To gain insight into the role of genomic alterations in breast cancer progression, we conducted a comprehensive genetic characterization of a series of four cell lines derived from MCF10A. MCF10A is an immortalized mammary epithelial cell line (MEC); MCF10AT is a premalignant cell line generated from MCF10A by transformation with an activated *HRAS* gene; MCF10CA1h and MCF10CA1a, both derived from MCF10AT xenografts, form well-differentiated and poorly-differentiated malignant tumors in the xenograft models, respectively. We analyzed DNA copy number variation using the Affymetrix 500 K SNP arrays with the goal of identifying gene-specific amplification and deletion events. In addition to a previously noted deletion in the *CDKN2A* locus, our studies identified *MYC* amplification in all four cell lines. Additionally, we found intragenic deletions in several genes, including *LRP1B* in MCF10CA1h and MCF10CA1a, *FHIT* and *CDH13* in MCF10CA1h, and *RUNX1* in MCF10CA1a. We confirmed the deletion of *RUNX1* in MCF10CA1a by DNA and RNA analyses, as well as the absence of the RUNX1 protein in that cell line. Furthermore, we found that RUNX1 expression was reduced in high-grade primary breast tumors compared to low/mid-grade tumors. Mutational analysis identified an activating *PIK3CA* mutation, H1047R, in MCF10CA1h and MCF10CA1a, which correlates with an increase of AKT1 phosphorylation at Ser473 and Thr308. Furthermore, we showed increased expression levels for genes located in the genomic regions with copy number gain. Thus, our genetic analyses have uncovered sequential molecular events that delineate breast tumor progression. These events include *CDKN2A* deletion and *MYC* amplification in immortalization, *HRAS* activation in transformation, *PIK3CA* activation in the formation of malignant tumors, and *RUNX1* deletion associated with poorly-differentiated malignant tumors.

## Introduction

Human breast cancer is thought to evolve via sequential genetic alterations from benign hyperplasia of mammary duct epithelial cells, through atypical ductal hyperplasia, to ductal carcinoma *in situ* (DCIS), invasive tumor confined to the breast, lymph node involvement, and eventually metastases to distant organs. A powerful cell culture model system for studying breast cancer progression is the MCF10A series of cell lines [1], [2], [3]. This system consists of multiple cancer cell lines derived from an immortalized mammary epithelial cell (MEC) line; these isogenic cell lines represent progression through stages of breast tumorigenesis in much the same manner as the *in vivo* human breast lesions reflect such a carcinogenic process. MCF10A is an immortalized mammary epithelial cell line; the premalignant cell line MCF10AT was generated by *HRAS* transformation of MCF10A; MCF10CA1h and MCF10CA1a, both derived from MCF10AT xenografts, form well-differentiated and poorly-differentiated malignant tumors in their respective xenograft models.

The MCF10A series of cell lines has frequently been used to study transformation activities of oncogenes and tumor suppressor genes. Recent studies include the investigation of the effects of *ERBB2* overexpression on cell invasion [4], deletion of *RB* on the epithelial-to-mesenchymal transition (EMT) [5], and *PCDH8* mutations on cell transformation [6]. In addition to studies of individual genes, several investigations have used array CGH to analyze DNA copy number variation of MCF10A [7], [8]. One such study found a deletion of the *CDKN2A* locus [8], which had also been noted in a previous cytogenetic analysis [9]. Another study using the multiplex ligation-dependent probe amplification assay containing 122 probe sequences uncovered gain in the *MYC* region in MCF10A [10]. A recent study reported a combined analysis of array CGH and cDNA microarray approaches for the MCF10A series cell lines [11]. Despite the progress made using the MCF10A model system, we still lack comprehensive knowledge of key genetic events that drive the progression of the MCF10A series of cell lines through the steps of immortalization, transformation, malignant tumor formation, and tumor metastasis. Using a combination of high density SNP arrays and mutation analysis by sequencing, we have identified sequential genetic alterations, both known and novel. These genetic changes include *CDKN2A* deletion and *MYC* amplification in immortalization, *HRAS* activation in transformation, *PIK3CA* activation in the formation of malignant tumors, and *RUNX1* deletion associated with poorly-differentiated malignant tumors.

## Results and Discussion

### Large-Scale Genomic Alterations in the MCF10A Series of Cell Lines

**Figure 1:**
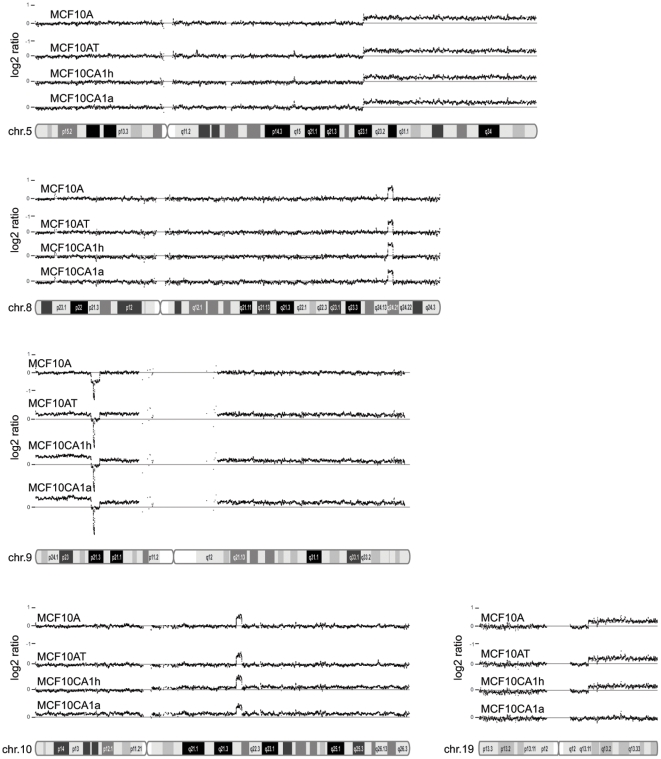
Figure 1 shows DNA copy number variation in chromosomes 5, 8, 9, 10, and 19. The graph was generated using the Affymetrix Genome Browser. Genomic position is displayed on the x-axis and log2ratio (tumor hybridization intensity normalized by diploid HapMap reference samples) is on the y-axis. DNA copy number gains at 5q23.1-qter, 8q24.21, and 10q22.1 are seen in all four cell lines. Homozygous deletion at the *CDKN2A* locus in chromosome 9p21.3 occurs in all four cell lines. Note that the homozygous deletion (400 kb) is embedded within a hemizygous deletion region (4 Mb). 19q13-qter copy number gain is observed in the MCF10A, MCF10AT, and MCF10CA1h cell lines.

To better understand the role of genetic alterations in breast cancer progression, we performed DNA copy number analysis using the Affymetrix 250 K Nsp and Sty SNP arrays. Genomic changes affecting chromosomes or sub-chromosomal regions are shown in Figure 1 for chromosomes 5, 8, 9, 10, and 19 (Plots for other chromosomes are available in Supplementary Figure S1, and the data are also summarized in Supplementary Figure S2A and Supplementary Table S1). Some of the observed alterations were present in most or all cell lines. For example, we detected copy number gain on the long arm of chromosome 5, spanning 5q23-qter region, in all 4 cell lines (Figure 1). This finding is in agreement with a duplication at the tip of 5q noted in a previous cytogenetic study [1]. Copy number gain was observed in the 19q13-qter region in MCF10A, MCF10AT, and MCF10CA1h (Figure 1). Other genomic alterations were restricted to subsets of the MCF10A cell line series. Among these were chromosome 20 trisomy (gain of whole chromosome) observed in the MCF10A cell line and the loss of the 13q21 region seen in the MCF10AT cell line (Supplementary Figures S1 and S2A).

From this analysis we found the global genomic structure of MCF10AT to be similar to that of MCF10A except for alterations in the following three regions: chromosome 9, 13q21, and chromosome 20 (Figure 1 and Supplementary Figures S1 and S2A and Table S1). These changes are relatively infrequent compared to the changes found only in MCF10CA1h. This observation suggests that relatively few additional genetic events were required beyond activation of the *HRAS* gene in order to achieve progression from immortal but otherwise normal MCF10A to pre-malignant MCF10AT. In contrast to the minimal number of genomic changes in the transition from the immortalized cell line MCF10A to the transformed cell line MCF10AT, MCF10CA1h, the malignant cancer cell line, acquired numerous genetic alterations relative to its MCF10AT precursor cell line, including both copy number gain and loss (Supplementary Figures S1 and S2A and Table S1). In contrast, at this chromosomal level the MCF10CA1a metastatic cells appear to recapitulate the gains and losses observed in the less aggressive cell lines. We found copy number gain in the 3p14-qter region in the two malignant cell lines (Supplementary Figures S1 and S2A and Table S1) and chromosome 9 trisomy in MCF10AT, MCF10CA1h, and MCF10CA1a cells (Figure 1). The copy number gains on chromosomes 3 and 9 have previously been observed in a cytogenetic study which described them as der(3)t(3;17)(p13;p12) and +9 trisomy [3]. There were numerous unique changes in the MCF10CA1h cell line, including the copy number gains on 1q, 2p, 14q12-q24, 15q11-q25, and 17q21-qter (Supplementary Figures S1 and S2A and Table S1), suggesting that a critical mutation which was acquired in the MCF10CA1h lineage may be associated with the observed genomic instability. One unique feature of the SNP chip is its capacity for detecting loss of heterozygosity (LOH). We identified numerous LOH regions (Supplementary Table S2). For example, MCF10CA1a cells showed complete loss of one allele for all SNPs on chromosomes 2 and 6 due to mitotic recombination or chromosomal non-disjunction (Supplementary Table S2 and Supplementary Figure S2B).

Next, we examined copy number changes affecting moderately sized genomic regions such as those involving a single cytogenetic band. We found copy number gain in 10q22.1 in all 4 cell lines (Figure 1). Interestingly, although duplication in the same region, noted as dup(10)(q22.1–22.2), was previously observed in this series of cell lines, these earlier observations reported this duplication to be restricted to MCF10CA1h and MCF10CA1a [3]. A possible explanation for this discrepancy is that dup(10q22) was present in a small fraction of the cells in MCF10A and MCF10AT, and these dup(10q22)-positive cells were expanded to constitute a major fraction in the MCF10A and MCF10AT cell populations used in the current study. If this interpretation is correct, the 19 genes located within the dup(10q22) region (Supplementary Table S3) may have provided a selective growth advantage. Another region with copy number gain in all 4 cell lines was found in 17p11 (Supplementary Figures S1 and S2A and Table S1). Additional copy number changes involving gain in 13q32 and loss in 21q11 were observed in all 4 cell lines (Supplementary Figures S1 and S2A).

### Focal Amplification and Deletion in the MCF10A Series of Cell Lines

Many of the large-scale genomic changes observed in our studies were also noted in previous cytogenetic studies. Our specific interest in this project, however, was to identify small genomic changes that affect regions a few hundred kilobases (kb) in size, with the goal of discovering novel oncogenes and tumor suppressor genes. To this end, we took advantage of the high marker density of the Affymetrix 500 K microarray to identify focal amplifications and deletions. We found *MYC* amplification at chromosome 8q24 (Figure 1, Table 1) in all 4 cell lines. The *MYC* amplification in MCF10A cells was only recently reported [10]. The method used by that study, however, did not define the boundaries of the amplified region at high resolution. Our analysis refined the region of amplification to a 2 Mb segment containing only four genes, *MYC*, *PVT1*, *FAM84B*, and *TMEM75*.

**Table 1:** Focal amplification and intragenic deletion and mutation in the MCF10 series cell lines.

chr	cytoband	gene	type of change and comments	MCF10A	MCF10AT	MCF10CA1h	MCF10CA1a
2	2q22.1	*LRP1B*	intragenic deletion			yes	yes
3	3p26.3	*CHL1, CNTN6*	focal deletion	yes	yes	yes	yes
3	3p14.2	*FHIT*	intragenic deletion			yes	
3	3q26.32	*PIK3CA*	H1047R mutation			yes	yes
8	8q24.21	*MYC, PVT1, FAM84B, TMEM75*	focal amplification; *MYC* is the likely target gene	yes	yes	yes	yes
9	9p21.3	*MTAP, CDKN2B, CDKN2A*	focal deletion; *CDKN2A* and *CDKN2B* are the likely target genes	yes	yes	yes	yes
21	21q22.12	*RUNX1*	intragenic deletion			yes, partial	yes

We observed a number of focal deletion events in the MCF10A series of cell lines. A deletion in 3p26.3, affecting the *CHL1* and *CNTN6* genes, was found in all 4 cell lines (Table 1). Our analysis also revealed homozygous deletion at the loci of *CDKN2A* and *CDKN2B* at chromosome 9p21.3 (Figure 1, Table 1). The homozygous deletion of *CDKN2A/B* was confirmed by PCR analyses using primers that span each exon of the two genes. We were able to detect PCR products on DNA templates isolated from normal cells but not from the MCF10A series of cell lines (data not shown). The *CDKN2A* locus homozygous deletion in MCF10A was already known [9]. The homozygous deletion of the *CDKN2A* locus was initially identified in a cytogenetic analysis [9] and was also observed in another study using the multiplex ligation-dependent probe amplification assay [10]. Our data narrowed down the deletion region to a 400 kb interval encompassing the *MTAP*, *CDKN2B*, and *CDKN2A* genes.

**Figure 2:**
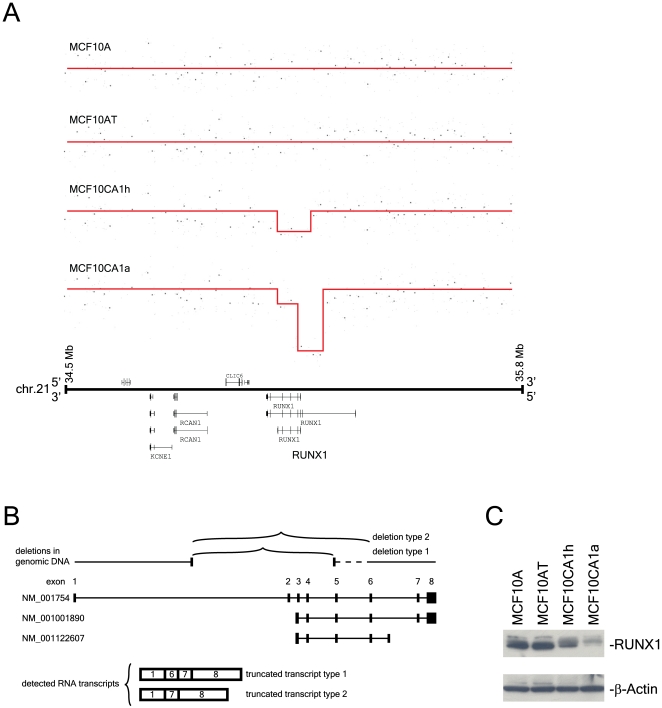
Intragenic RUNX1 deletions in the MCF10A series of cell lines. **Figure 2A illustrates intragenic DNA deletion and partial deletion in MCF10CA1a and MCF10CA1h, respectively.** The deletions span the promoter regions of two of the transcripts. Note that the transcripts are oriented from right to left in this figure. **Figure 2B summarizes altered transcripts detected in MCF10CA1a cells due to indicated genomic deletions.** Deletion structure is illustrated here based on the data shown in Figure 2A. Three alternative transcripts of the *RUNX1* gene are shown here. Truncated transcripts involving removal of exons 2–5 (truncated type 1) or exons 2–6 (truncated type 2). **Figure 2C shows Western blot analysis of the RUNX1 protein in the MCF10A series of cell lines.** RUNX1 protein expression was analyzed by Western blot using anti-RUNX1 antibody. The β-actin staining shows a similar level of protein loading in all lanes. Note the dramatic reduction of the RUNX1 protein in MCF10CA1a cells. MCF10CA1h cells show a reduced level of RUNX1 protein.

We also observed intragenic deletions within several genes which potentially function as breast cancer tumor suppressor loci. These included deletions which disrupted *LRP1B* in MCF10CA1h and MCF10CA1a (Supplementary Figure S2C), *FHIT* and *CDH13* in MCF10CA1h (data not shown), and *RUNX1* in MCF10CA1a and to a lesser extent in MCF10CA1h (Figure 2A, Table 1). We validated each of these intragenic deletions by qPCR (quantitative PCR) (data not shown).

### Molecular Characterization of *RUNX1* in the MCF10A Series of Cell Lines

We undertook a detailed molecular analysis of the *RUNX1* gene since it was deleted predominantly in MCF10CA1a, which forms poorly-differentiated malignant tumors in xenografts. *RUNX1*, also called *AML1*, encodes the alpha subunit of the core binding factor (CBF), which binds to the core element of many enhancer and promoter DNA regions. Chromosomal translocations involving this gene are commonly associated with several types of leukemia (See [12], [13] for recent reviews). However, to date no study of *RUNX1* in breast cancer has been reported.

Three wild type transcripts are encoded by the *RUNX1* gene, one long and two short transcripts. The long transcript differs from the two short transcripts in terms of its transcription start, whereas the two short transcripts differ from each other in their termination sites (Figure 2B). To characterize any potential structural changes in the long transcript, we carried out RT-PCR using a pair of primers in exon1 and exon7, followed by sequencing the RT-PCR products. Intragenic deletion in *RUNX1*, observed in the genomic DNA in the MCF10CA1a cell line, is expected to generate truncated transcripts (Figure 2B). In agreement with the predicted transcript structure based on the genomic DNA alterations that we observed, all RT-PCR products showed a deletion of exons 2–5, with a subset of products exhibiting an additional deletion extending through exon 6 (Figure 2B). Both aberrant transcripts maintain the RUNX1 open reading frame, but result in the loss of the RUNT domain, which is critical for DNA binding and protein dimerization. The same deletions were also observed in a fraction of RT-PCR products generated from MCF10CA1h cells, which is consistent with the partial deletion in DNA copy number suggested in the analysis shown in Figure 2A. Using a primer specific for the short transcripts in qRT-PCR experiments, we showed that expression of these transcripts was normal in MCF10A and MCF10AT cells but reduced in MCF10CA1h cells and nearly absent in MCF10CA1a cells (Supplementary Figure S3A).

We then characterized RUNX1 protein expression by Western blot analysis. The RUNX1 protein was nearly absent in the MCF10CA1a cell line (Figure 2C), in agreement with the absence of the full-length transcript. The RUNX1 protein level was reduced in the MCF10CA1h cell line when compared to the level in the MCF10AT cell line, which was the precursor cell line to both MCF10CA1h and MCF10CA1a. Thus, this protein expression pattern is similar to that observed in RNA expression experiments (Supplementary Figure S3A). Our results suggest that loss of RUNX1 protein due to intragenic deletion may be a key step in the development of the malignant breast cancer phenotype.

**Figure 3:**
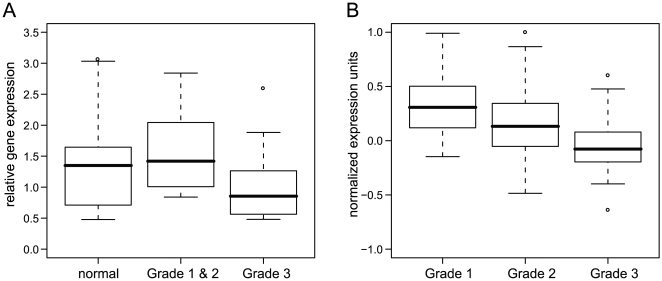
Expression of RUNX1 gene in primary breast tumors. **Figure 3A shows qRT-PCR analysis of the RUNX1 mRNA expressions in primary breast tumors and the adjacent normal samples.** 25 of 29 our analyzed tumors have histological grade information. High histological grade tumors (n = 16) have significant reduction of RUNX1 expression compared to low/mid grade tumors (n = 9) or to the adjacent normal samples (n = 11). Data are summarized by box plots. The box represents gene expression values between 1^st^ and 3^rd^ quartiles; the line denotes median. Gene expression difference between high-grade (grade 3) and low/mid-grade (grade 1 plus grade 2) is significant (pvalue = 0.044, by t-test). **Figure 3B shows RUNX1 mRNA expression in Ivshina breast microarray dataset.** Ivshina breast dataset consists of 289 tumors (68 Grade 1, 166 Grade 2, and 55 Grade 3). Affymetrix U133A array expression data showed progressive reduction of RUNX1 gene (reporter: 210365_at) from Grade 1 to Grade 2 to Grade 3. The progressive reduction of RUNX1 expression in higher grade tumors is highly significant (p-value = 3×10^−13^ by linear regression analysis). Oncomine™ (Compendia Bioscience, Ann Arbor, MI) was used for this analysis.

We recently reported DNA copy number analysis for 161 primary breast tumors using the Affymetrix 500 K SNP arrays [14]. Using this dataset, we analyzed DNA copy number of *RUNX1* in the primary tumors. Our analysis found no evidence of genomic alteration of the *RUNX1* gene. However, tumor heterogeneity poses a significant challenge to the detection of intragenic deletions that are present in only a portion of a tumor sample. To investigate gene expression of *RUNX1*, we performed quantitative RT-PCR (qRT-PCR) analysis for 29 primary tumors from which we were able to isolate high quality RNA. We also did qRT-PCR for 11 RNA samples isolated from normal tissues adjacent to the tumors. We found that RUNX1 expression was down-regulated significantly in high-grade tumors compared to low/mid-grade tumors (Figure 3A, p-value = 0.044 by t-test). Thus, our qRT-PCR analysis of RUNX1 expression in primary tumors corroborated the study in the MCF10A series of cell lines and supported the idea that *RUNX1* is a potential tumor suppressor gene involved in carcinogenic progression in this model of breast cancer.

To extend RUNX1 expression analysis in primary tumors, we performed data mining of Oncomine database (Compendia Bioscience, Ann Arbor, MI). We found that RUNX1 expression was progressively reduced from low- to mid- to high-grade tumors in multiple datasets that include Ivshina breast dataset (n = 289, Figure 3B) and Lu breast dataset (n = 129, Supplementary Figure S3B). The reduction of RUNX1 expression in high-grade tumors is highly significant (p-value = 3×10^−13^ for the Ivshina dataset and p-value = 2.5×10^−9^ for the Lu dataset by linear regression model analyses). Thus, the results provide further support that *RUNX1* is a candidate tumor suppressor gene involved in breast cancer development.

### Mutational Analysis of Known Oncogenes and Tumor Suppressor Genes in the MCF10A Series of Cell Lines

**Figure 4:**
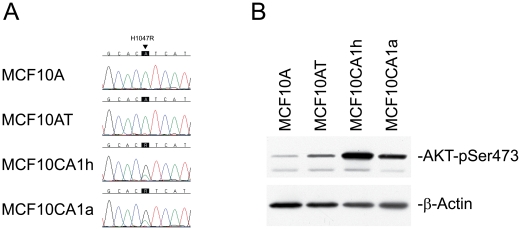
Identification of *PIK3CA* mutation in the MCF10CA1h and MCF10CA1a cells. **Figure 4A shows a segment of the sequencing chromatogram of** ***PIK3CA*** **.** MCF10A and MCF10AT cells have a wild type A-allele marked by an arrow. In contrast, MCF10CA1h and MCF10CA1a cells exhibit predominance of the mutant G-allele in addition to the wild type A-allele. The mutation results in an amino acid change from His to Arg at 1047 (H1047R). **Figure 4B shows increased phosphorylation of AKT1 Ser473 in the MCF10CA1h and MCF10CA1a cells.** An antibody targeting phosphorylated Ser473 in AKT1 recognized specifically the phosphorylated form of the protein, which was dramatically increased in cells containing the *PIK3CA* activating mutation, i.e. the MCF10CA1h and MCF10CA1a cells. β-actin served as a control for protein loading.

To identify subtle changes such as point mutations, we conducted sequence analysis of the tumor suppressor genes and oncogenes that are most commonly mutated in cancer, including *TP53*, *PIK3CA*, *AKT1*, *HRAS*, and *BRAF*. We carried out the mutation analysis for the following regions of these five genes: *TP53* (exons 4–9), *PIK3CA* (exons 10 and 21), *BRAF* (exons 11 and 15), *AKT1* (exon 3), and *HRAS* (exons 1 and 2). Besides the G12V HRAS mutation used in the transformation experiment to derive the premalignant cell line MCF10AT from MCF10A, the only mutation we detected was *PIK3CA* H1047R, which is known to be the most common activating mutation in the *PIK3CA* gene in human cancer [15], [16]. We observed this *PIK3CA* mutation to be present specifically in MCF10CA1h and MCF10CA1a cells (Figure 4A, wild type A-allele changed to mutant G-allele) but to be absent in the precursor MCF10AT cell line. Quantitative estimation of the mutant versus wild type allele indicated a ratio of 2 to 1, suggesting that the mutant allele was present on the chromosome that was duplicated in der(3)t(3;17). AKT1 is often phosphorylated at Ser473 and Thr308 when PIK3CA is activated. Therefore, we evaluated AKT1 Ser473 and Thr308 phosphorylation in the 4 cell lines of the MCF10A series. As expected, we saw a high level of phosphorylation of AKT1 Ser473 in MCF10CA1h and MCF10CA1a cells (Figure 4B) which have the activating PIK3CA mutation. AKT1 Thr308 phosphorylation was also elevated in the two malignant cell lines (data not shown). Our results are consistent with the findings of two recent papers which reported increased phosphorylation of AKT1 in these malignant cells; one of these reports also noted the presence of the PIK3CA H1047R mutation in the malignant cells [17], [18]. Our findings, together with those of the previous studies, suggest that this *PIK3CA* activating mutation could be the critical genetic change driving the malignant phenotype of MCF10CA1h and MCF10CA1a cells.

### The Effect of Copy Number on Global Gene Expression

**Figure 5:**
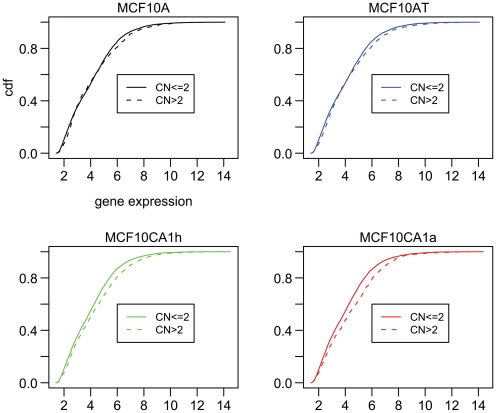
The effect of chromosome copy number gain on gene expression in the MCF10A series of cell lines. For each cell line, genes are grouped into those showing copy number (CN) greater than 2 (CN >2, dashed lines) and those showing copy number equal to or less than 2 (CN ≤2, solid lines). Expression levels of genes are indicated along the X-axis. The Y-axis represents the cumulative distribution function (cdf), which describes cumulative probability of gene expression less than or equal to the threshold level indicated by the value on X-axis.The data depicted in this figure indicate show largedifference between the expression levels for genes with copy number gain (CN >2) versus those with no gain (CN ≤2) only in the two malignant cell lines, MCF10CA1h and MCF10CA1a.

To study the impact of chromosome copy number gain on global gene expression, we performed gene expression profiling for the 4 cell lines using the Affymetrix Human Gene Array 1.0 ST. We first mapped all genes in the chromosome regions with copy number gain using the Affymetrix 500 K SNP array. For each of the 4 cell lines, the regions that showed copy number gain on the 500 K SNP array overlapped the genes that were interrogated for expression on the Human Gene Array to different degrees. Thus, for the MCF10A cell line only 6% of the 19,734 genes interrogated by the expression array fall into regions that showed copy number gain on the 500 K SNP chip. In contrast, 8%, 23%, and 16% of these 19,734 genes are represented in copy number gain regions in the cell lines of MCF10A, MCF10AT, and MCF10CA1h, respectively. Comparisons between genomic regions with copy number gain (Figure 5, dashed lines labeled as “CN>2”) to those without gain (Figure 5, solid lines labeled as “CN< = 2”, which includes copy number neutral and copy number loss, the latter being a rare event, see Supplementary Figure S2A) showed increased expression levels for genes located in the regions with DNA copy number gain. The increased gene expression in the two malignant cell lines is highly significant (p-value = 2×10^−9^ for MCF10CA1h and p-value = 2×10^−12^ forMCF10CA1a, t-test). Our results contrast with those of Marella et al. [11], which showed a lack of correlation between gene expression and copy number alterations. Their conclusion was based on the analysis of 40 genes in the MCF10CA1a cells; they found 13 up and 18 down regulated genes in genomic gain regions whereas 3 down and 6 up regulated genes in the genomic loss regions. The small number of genes analyzed in that study may not reflect the global pattern that we have detected in terms of overall gene expression up-regulation in genomic copy number gain regions in the MCF10CA1h and MCF10CA1a cell lines. The genes that showed at least 4-fold difference in some of the pair-wise comparisons among the MCF10A, MCF10AT, MCF10CA1h, and MCF10CA1a cell lines are included in Supplementary Tables S4, S5, S6, S7.

Gene expression profiling of human breast cancers has identified a number of distinct molecular subtypes with different biological properties and prognoses [19]. The MCF10A parental cell line clusters most closely with human breast cancer cell lines of the “Basal B” subtype in expression profiling [7], and it lacks functional estrogen or progesterone receptors [20]. The transformed MCF10AT cells form hyperplastic ductal structures on xenografting that have both myoepithelial and luminal components, suggesting that the MCF10A parental line has properties of a bipotential breast epithelial progenitor [2]. While MCF10CA1h tumors have a mixed histology that includes well-differentiated regions with weak expression of estrogen receptor, the more malignant MCF10CA1a cells form poorly differentiated tumors that lack estrogen receptor [21]. Furthermore, in our present study, which has focused on copy number changes, we have shown that the *HER2* locus is not amplified in any of the four cell lines. While recognizing that there are limitations inherent in any model, by the criteria of hormone receptor negativity and lack of amplification of the *HER2* locus, as well as by clustering of the parental MCF10A cell line with the Basal B subgroup by transcriptional profiling, the MCF10 progression series appears to most closely model the development of the poor prognosis “basal-like” subgroup of human breast cancers.

In conclusion, our genome-wide analyses have uncovered sequential mutation events, both known and novel, which delineate breast tumor progression. These events include *CDKN2A* deletion and *MYC* amplification in immortalization, *HRAS* activation in transformation, *PIK3CA* activation in the formation of malignant tumors, and *RUNX1* deletion associated with poorly-differentiated malignant tumors.

## Materials and Methods

### DNA, RNA Isolation, and Microarray Experiments

DNAs were isolated using Blood and Cell Culture midi Kit (Qiagen, Inc., Valencia, CA, USA) and RNAs were isolated using RNeasy mini kit (Qiagen). We followed the manufacturer's protocol to perform DNA copy number analysis using the Affymetrix 500 K SNP arrays and gene expression profiling experiments using Human Gene 1.0 ST arrays.

### cDNA Synthesis, RT-PCR, Cloning Sequencing and Quantitative RT-PCR

Total RNA was treated with DNase (Ambion, Austin, TX, USA), and cDNA was synthesized using SuperScript® III reverse transcriptase (Invitrogen, Carlsbad, CA, USA) with random primers following the manufacturer's suggested protocol. PCR was carried out using primers specific for exon 1 and exon 7 of the RUNX1 transcript (NM_001754). PCR products were checked by an agarose gel electrophoresis and then cloned into pCR-TOPO TA cloning vector (Invitrogen). Plasmids were purified using the mini prep kit (Qiagen) and were subjected to sequencing reactions using the ABI BigDye Terminator BDT 3.1 (Applied Biosystems, Foster City, CA, USA). The sequencing reaction was carried out at 96°C for 10 sec, 50°C for 5 sec, 60°C for 2 min for 25 cycles, with the M13 forward or reverse primers, and the reaction products were analyzed using the 3730 XL DNA Analyzer (Applied Biosystems). Quantitative RT-PCR for RUNX1 was carried out using Taqman gene expression master mix (Applied Biosystems) and custom-synthesized oligos of RUNX1-forward (GATTAGCTGAAGATCTCTGAAACGCT), RUNX1-reverse (GTACTTGTCATGTTCTCTGTTCTCTCA) and FAM-MGB labeled Taqman probe (CAGTGCAGAAAATTC). PCR reaction was performed at 95°C for 15 sec and 60°C for 1 min for 40 cycles. The reaction products were analyzed with ABI Prism 7900HT sequence detection system (Applied Biosystems). Taqman gene expression analysis against PPIA gene (Applied Biosystems, Hs99999904_m1) was used as a control for normalizing the amount of RNA in the reaction.

### Mutation Analysis

Genomic DNA spanning exon and nearby intron sequences was amplified using the primers described in Supplementary Table S8. PCR was carried out in a 20 ul reaction mixture containing 1X Buffer (Applied Biosystems), 1.5 mM Mg^++^, 0.2 mM dNTP, 0.5 uM primers, 5 ng genomic DNA, and 1 U Taq DNA polymerase (Applied Biosystems). Initial denaturing was at 95°C for 2 min; then 40 cycles of 95°C for 45 sec, 60°C for 30 sec, and 72°C for 60 sec; this was followed by extension at 72°C for 10 min. The PCR products were subjected to sequencing reactions using the ABI BigDye Terminator BDT 3.1 (Applied Biosystems) and the reaction products were analyzed using the 3730 XL DNA Analyzer (Applied Biosystems).

### Cell Culture and Western Blot Analysis

The MCF10A series cell lines were described in a previous paper [1], [2], [3], [22]. The MCF10AT subline used here was MCF10AT1k.cl2 (herein referred to as MCF10AT), which was the specific clone that was used to derived the tumorigenic variants, MCF10CA1h and MCF10CA1a.cl1 (referred to here as MCF10CA1a) [3]. MCF10A and MCF10AT cells are maintained in DMEM/F12 medium supplemented with 5% horse serum, 10 µg/ml insulin, 20 ng/ml EGF, 0.5 µg/ml hydrocortisone, and 100 ng/ml cholera toxin. Culture medium for MCF10CA1h and MCF10CA1a cell lines are DMEM/F12 plus 5% horse serum. Cells for microarray gene expression analysis were cultured in DMEM/F12 supplemented with serum replacement 1 (Sigma, St. Louis, MO, USA). For Western blot analyses, cells grown to 70–80% confluence in regular growth medium (for RUNX1 analysis) or in DMEM/F12 supplemented with serum replacement 1 (Sigma) (for AKT1 phosphorylation analysis) was lysed and collected using M-PER mammalian protein extraction reagent (Thermo Fisher Scientific, Rockford, IL, USA). 30 ug of protein was loaded onto a tris-glycine SDS PAGE gel (Invitrogen) and semi-dry transferred to a nitrocellulose membrane using iBlot (Invitrogen). Dilutions for primary antibodies were 1∶250 for RUNX1 (sc-101146, Santa Cruz Biotechnology, Santa Cruz, CA, USA) and 1∶500 for AKT1 Ser473 (M3628, Dako, Carpinteria, CA, USA) both in 5% milk/TBST. Antibody targeting at β-actin (A5441, 1∶5,000 dilution; Sigma) wasused to assess equivalence of protein loading. Goat anti-rabbit HRP antibody or rabbit anti-mouse HRP antibody was used at a 1∶5000 dilution and the signals were detected by ECL.

### Data analysis

Microarray data were first normalized using the gtype-probeset-genotype package included in Affymetrix Power Tools version 1.85. Genomic DNA was characterized using the Affymetrix 250 K Nsp and Sty arrays. The Gene Expression Omnibus (GEO) accession number for these array data is GSE19920. Microarray data were normalized against 127 CEPH lymphoblastoid cell lines by quantile normalization. The output of quantile normalization is log2ratio of sample signal intensity to reference signal intensity for each probeset. The Affymetrix CNAT4.0 software produces a CN (copy number) estimate as log2ratio and a CN state prediction based on a Hidden Markov Model. For global analysis of copy number changes, we used bandwith of 100 kb, transition_decay at 1e-7, and no outlier_smoothing. For focal amplification/deletion, we used the bandwith of 1 kb. The CNstate ranges from 0 to 4: normal CN corresponds to CNstate 2; CNstates 0 and 1 indicate copy number loss; CNstates 3 and 4 correspond to copy number gain. For gene-level copy number estimation, we calculated the average log2ratio for all the probesets mapped within a gene, between the transcription start and termination sites of the gene. The gene-level log2ratio was used to identify gene amplification/deletion in each cell line. We used Affymetrix Expression Console Software to extract expression values generated from Human Gene 1.0 ST array experiments. All the statistical analyses were conducted using the R package.

## Supporting Information

Figure S1 DNA copy number analysis of the MCF10A series of cell lines. DNA copy numbers for indicated chromosomes are shown. The graph was generated using the Affymetrix Genome Browser. Genomic position is displayed on the x-axis and log2ratio (tumor hybridization intensity normalized by diploid HapMap reference samples) is on the y-axis.
(0.21 MB PDF)

Figure S2 A. DNA copy number variation is shown with a heatmap. The tick marks on the left show boundaries between the two adjacent chromosomes. The color scale indicated at the bottom depicts DNA copy number from 0 to > = 4.0 (blue represents the maximum deletion; tan represents the maximum amplification). B. Genetic alteration at chromosomes 2, 3 and 6 in MCF10CA1a cells. The graph was generated using Partek genomic suite software. DNA copy number (CN) is displayed as log2 ratio against the diploid control (in red). Allelic ratio (displayed in blue) represents the ratio of A-allele CN value divided by (A-allele + B-allele) CN value. Cytogenetic ideograms of chromosomes are displayed on the x-axes. Loss of heterozygosity (LOH) due to either mitotic recombination or chromosomal non-disjunction in chromosomes 2 and 6 are evident from the allele specific analysis. Chromosome 3 displays chromosomal gain (trisomy) in MCF10CA1a cells. Note that most of the normal p-arm on chromosome 3 has log2(CN ratio) value of 0 (diploid) with an allelic ratio of 0.5. C. Intragenic DNA deletion within the LRP1B locus in the MCF10CA1h and MCF10CA1a cell lines. Intragenic deletion in LRP1B is present in the MCF10CA1a and MCF10CA1a cell lines. Note that the transcript is oriented from right to left in this figure.
(2.45 MB PDF)

Figure S3 A. Quantitative RT-PCR analysis of the RUNX1 short transcripts. A primer was designed within the 5 prime end of exon 1 in the two short transcripts (NM_001001890 and NM_001122607) that is absent in the long transcript (NM_001754). Quantitative qPCR was carried out using Power SYBR Master Mix (Applied Biosystems) and RUNX1_variant2_forward and RUNX1_variant2_reverse primers at 95°C for 15 sec and 60°C for 1 min for 40 cycles. The reaction products were analyzed with ABI Prism 7900HT sequence detection system (Applied Biosystems). Taqman gene expression analysis against PPIA gene (Applied Biosystems, Hs99999904_m1) was used as a control for normalizing the amount of RNA in the reaction. The short transcripts are expressed in MCF10A, MCF10AT, and MCF10CA1h but absent in MCF10CA1a. Data are shown as mean +/− standard deviation of triplicate measurments. B. RUNX1 gene expression analysis using Oncomine database Breast cancer dataset showed reduced RUNX1 gene expression in high grade tumor. Lu breast dataset (n = 129) on Affymetrix U133 Plus 2.0 array also showed reduced RUNX1 (reporter: 209360_s_at) gene expression in Grade 3 tumor (n = 64) compared to lower grade tumor (n = 15 for Grade 1 and n = 16 for Grade 2). Represented in the box plots are, maximum and minimum values, whiskers at 90th and 10th percentile, boxes at 75th and 25th percentile, and the median. Oncomine (Compendia Bioscience, Ann Arbor, MI) was used for analysis and visualization.
(0.80 MB PDF)

Table S1 This table summarizes global DNA copy number changes at chromosomal and sub-chromosomal levels for each of the four cell lines.
(0.03 MB XLS)

Table S2 This table contains the genomic regions with LOH that were detected in MCF10AT, MCF10CA1h, and MCF10CA1a cells. LOH was computed based on the comparisons between each of the 3 cell lines with the MCF10A cell line.
(0.03 MB XLS)

Table S3 This table has the list of genes that are mapped in 10q22.1 CN gain region.
(0.03 MB XLS)

Table S4 Genes display 4-fold difference in expression levels between MCF10A andMCF10AT. The values are log2(MCF10AT/MCF10A). The genes included here have log2ratio >2 or log2ratio <−2.
(0.04 MB XLS)

Table S5 Genes display 4-fold difference in expression levels between MCF10AT and MCF10CA1h. The values are log2(MCF10CA1h/MCF10AT). The genes included here have log2ratio >2 or log2ratio <−2.
(0.05 MB XLS)

Table S6 Genes display 4-fold difference in expression levels between MCF10AT and MCF10CA1a. The values are log2(MCF10CA1a/MCF10AT). The genes included here have log2ratio >2 or log2ratio <−2.
(0.05 MB XLS)

Table S7 Genes display 4-fold difference in expression levels between MCF10CA1h and MCF10CA1a. The values are log2(MCF10CA1a/MCF10CA1h). The genes included here have log2ratio >2 or log2ratio <−2.
(0.05 MB XLS)

Table S8 This table has the primer sequences used in PCR experiments and mutation analyses for the TP53, PIK3CA, AKT1, HRAS, and BRAF genes.
(0.03 MB XLS)
