# Supplementary material for: Delineating Genetic Alterations for Tumor Progression in the MCF10A Series of Breast Cancer Cell Lines
Source: PLoS One. 2010 Feb 15;5(2):e9201. doi: 10.1371/journal.pone.0009201 (PMC2821407; doi:10.1371/journal.pone.0009201)
Supplement: Figure S1 — DNA copy number analysis of the MCF10A series of cell lines. DNA copy numbers for indicated chromosomes are shown. The graph was generated using the Affymetrix Genome Browser. Genomic position is displayed on the x-axis and log2ratio (tumor hybridization intensity normalized by diploid HapMap reference samples) is on the y-axis. (0.21 MB PDF) [file pone.0009201.s001.pdf]

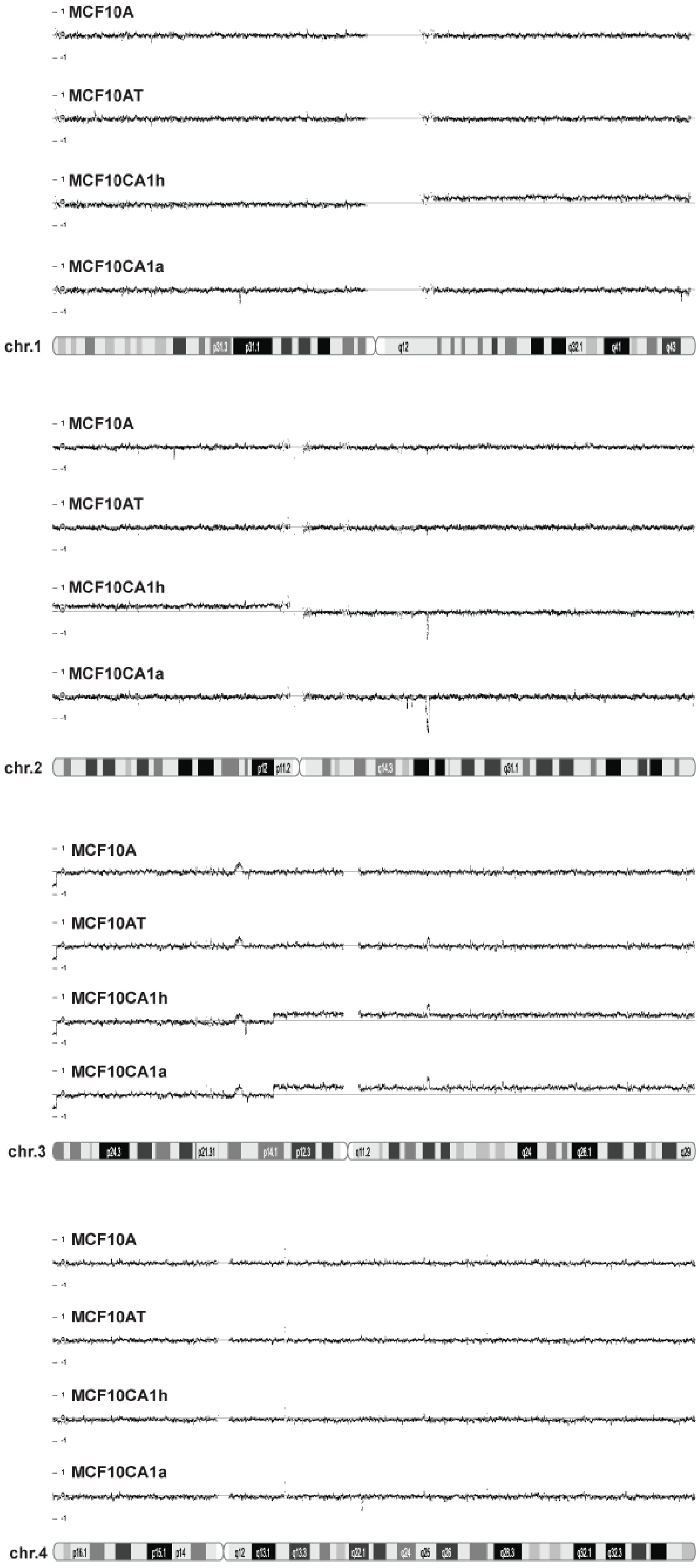
MCF10A
MCF10AT
MCF10CA1h
MCF10CA1a
chr.1
MCF10A
MCF10AT
MCF10CA1h
MCF10CA1a
chr.2
MCF10A
MCF10AT
MCF10CA1h
MCF10CA1a
chr.3
MCF10A
MCF10AT
MCF10CA1h
MCF10CA1a
chr.4

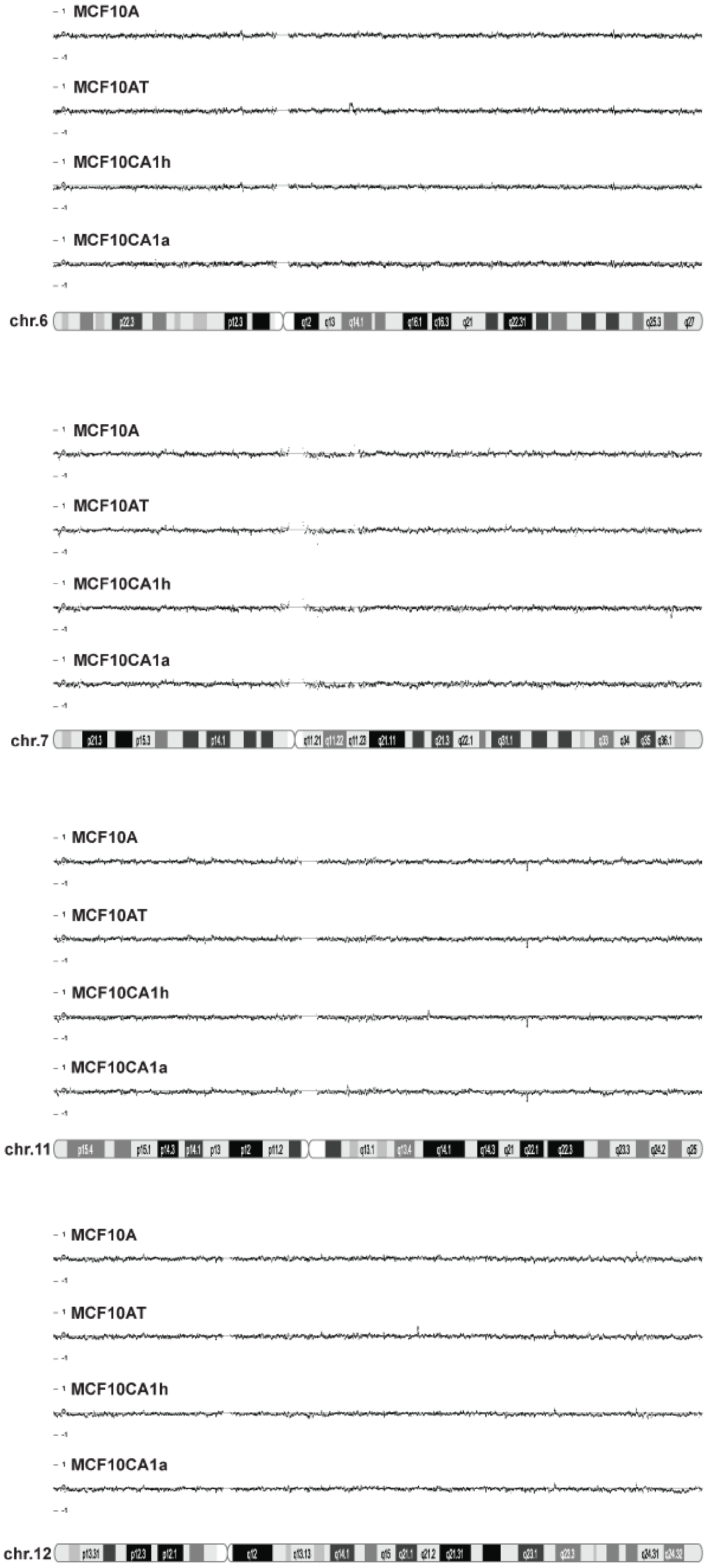

MCF10A
MCF10AT
MCF10CA1h
MCF10CA1a
chr.6
MCF10A
MCF10AT
MCF10CA1h
MCF10CA1a
chr.7
MCF10A
MCF10AT
MCF10CA1h
MCF10CA1a
chr.11
MCF10A
MCF10AT
MCF10CA1h
MCF10CA1a
chr.12

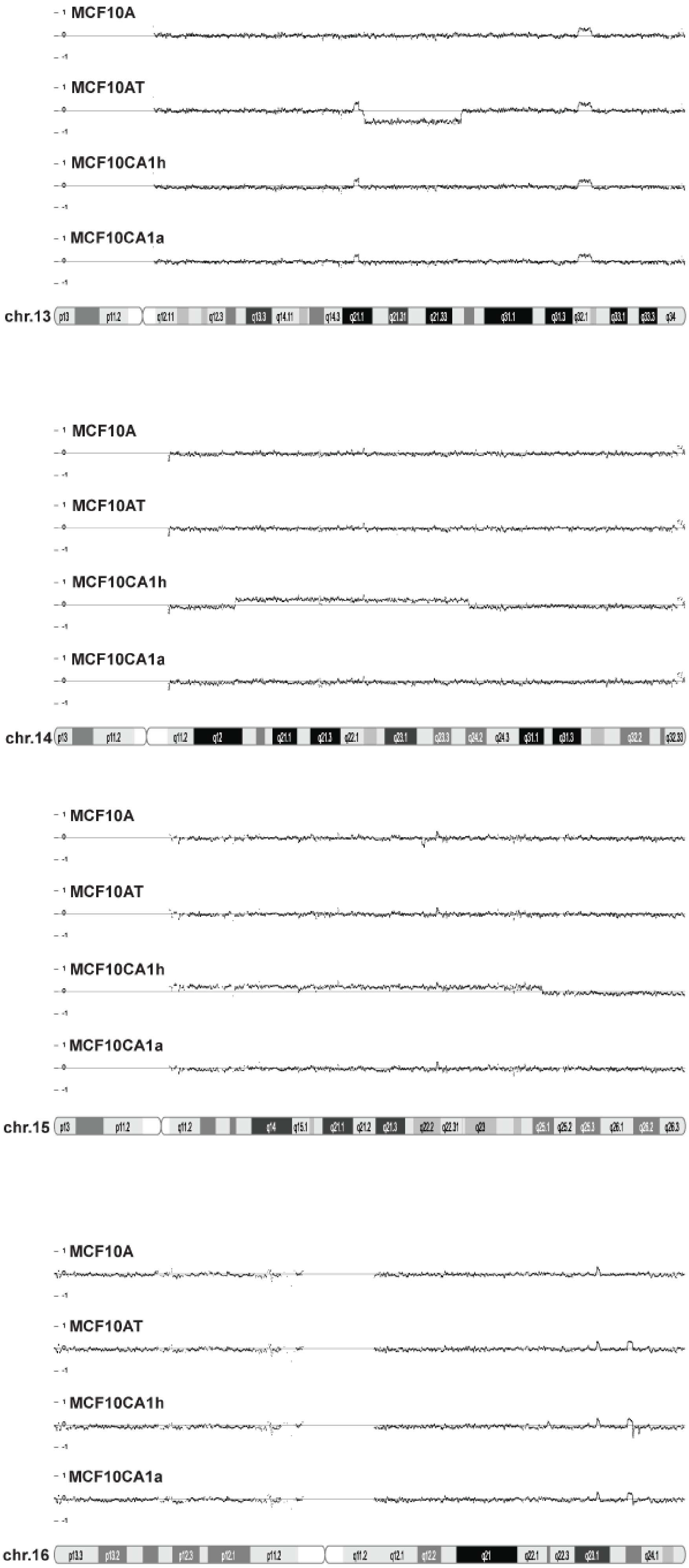
MCF10A
MCF10AT
MCF10CA1h
MCF10CA1a
chr.13
MCF10A
MCF10AT
MCF10CA1h
MCF10CA1a
chr.14
MCF10A
MCF10AT
MCF10CA1h
MCF10CA1a
chr.15
MCF10A
MCF10AT
MCF10CA1h
MCF10CA1a
chr.16

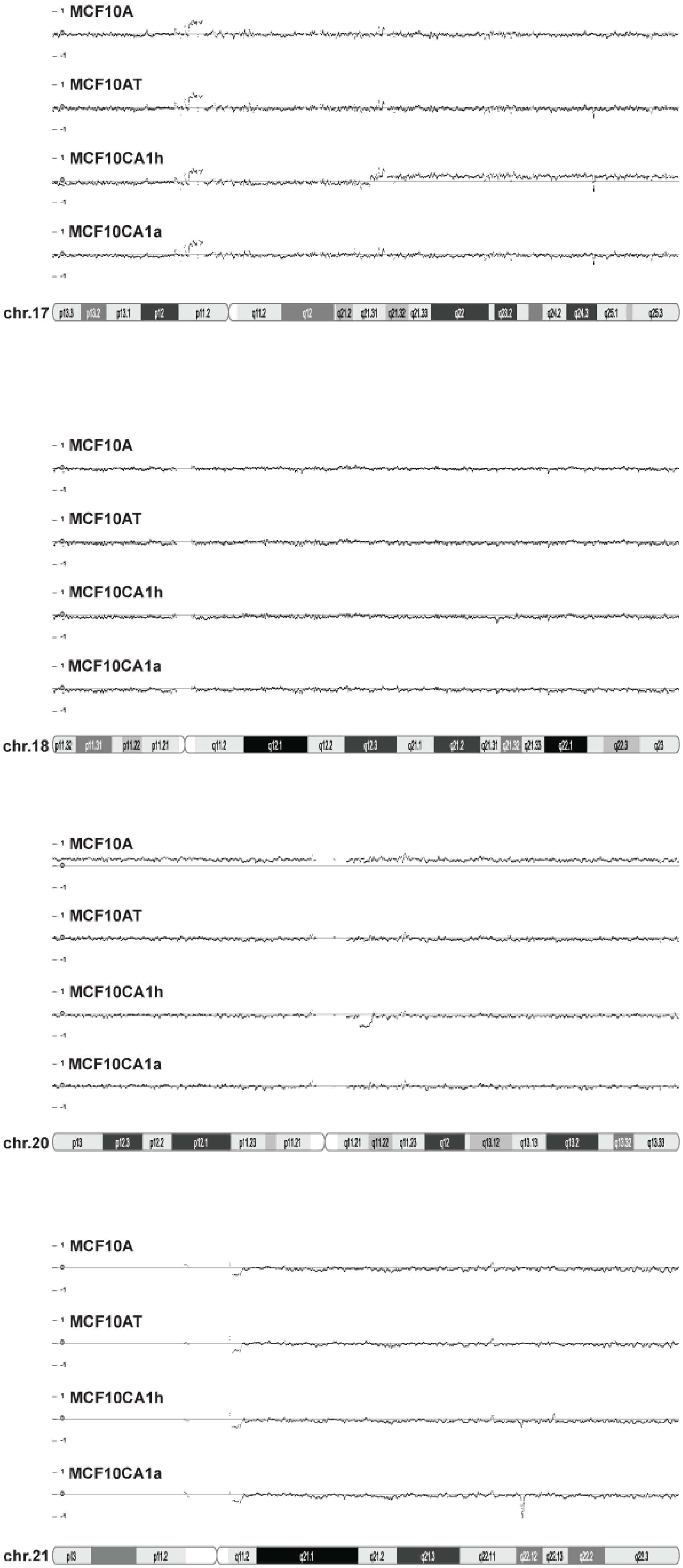
MCF10A
MCF10AT
MCF10CA1h
MCF10CA1a
chr.17
MCF10A
MCF10AT
MCF10CA1h
MCF10CA1a
chr.18
MCF10A
MCF10AT
MCF10CA1h
MCF10CA1a
chr.20
MCF10A
MCF10AT
MCF10CA1h
MCF10CA1a
chr.21

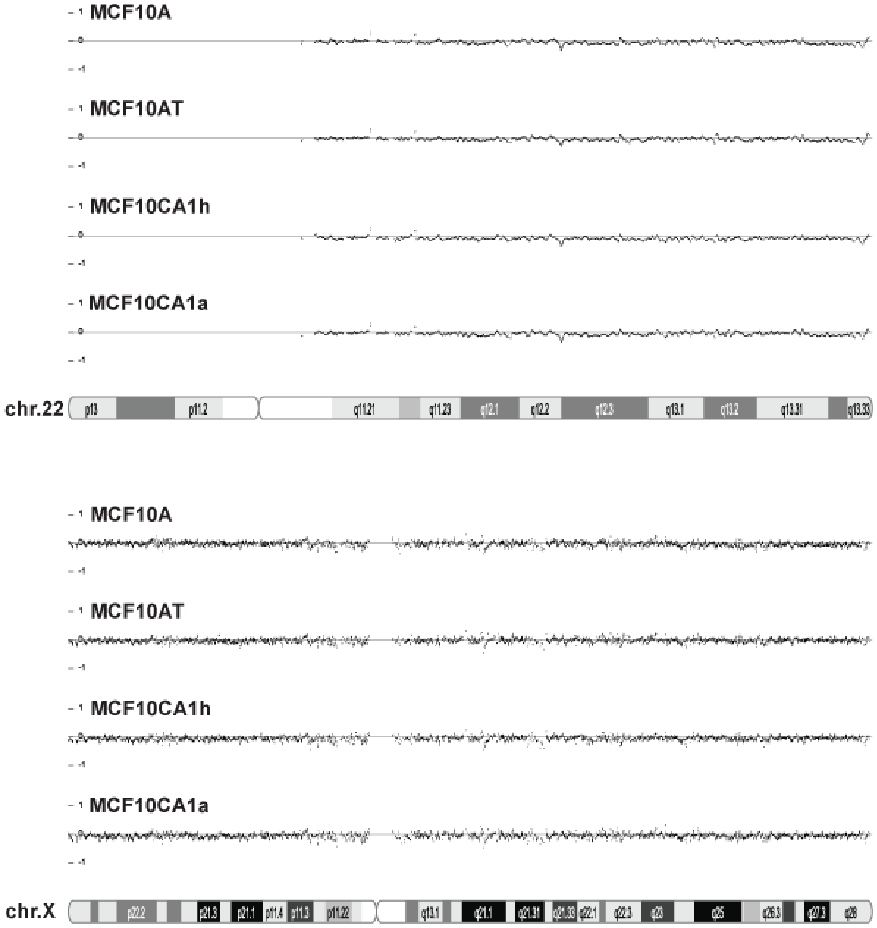
MCF10A
MCF10AT
MCF10CA1h
MCF10CA1a
chr.22
MCF10A
MCF10AT
MCF10CA1h
MCF10CA1a
chr.X
