# Supplementary figures and images for: Delineating Genetic Alterations for Tumor Progression in the MCF10A Series of Breast Cancer Cell Lines
Source: PLoS One. 2010 Feb 15;5(2):e9201. doi: 10.1371/journal.pone.0009201 (PMC2821407; doi:10.1371/journal.pone.0009201)

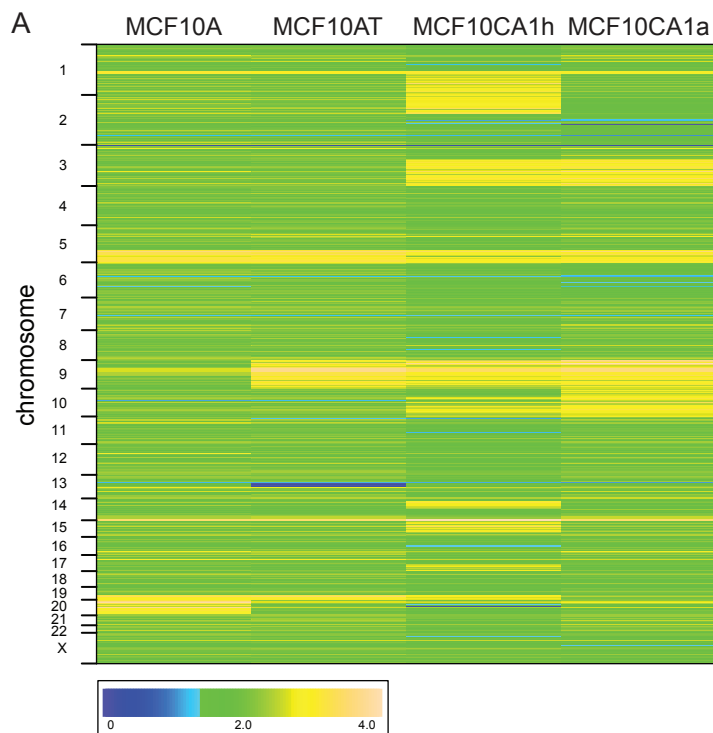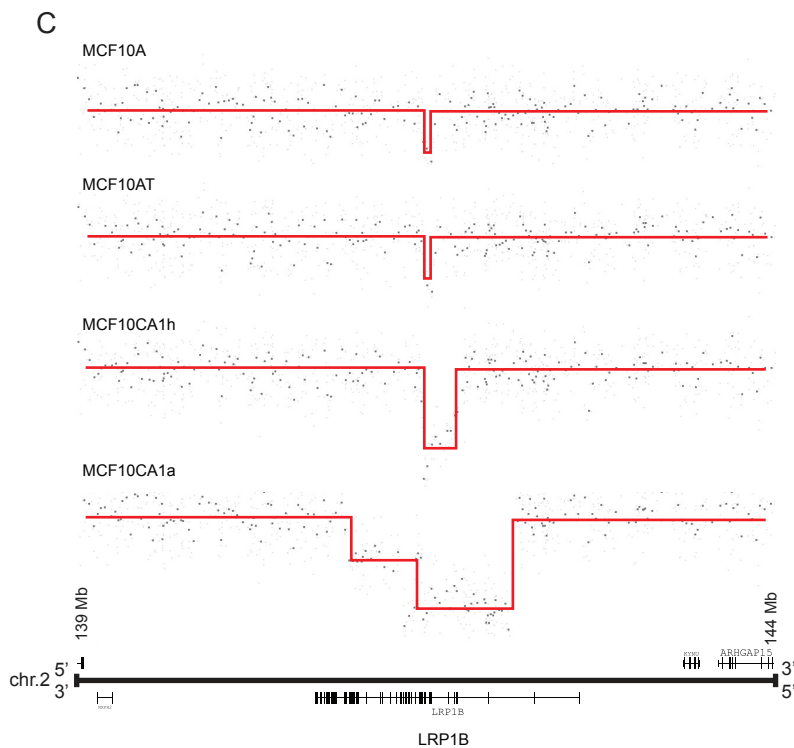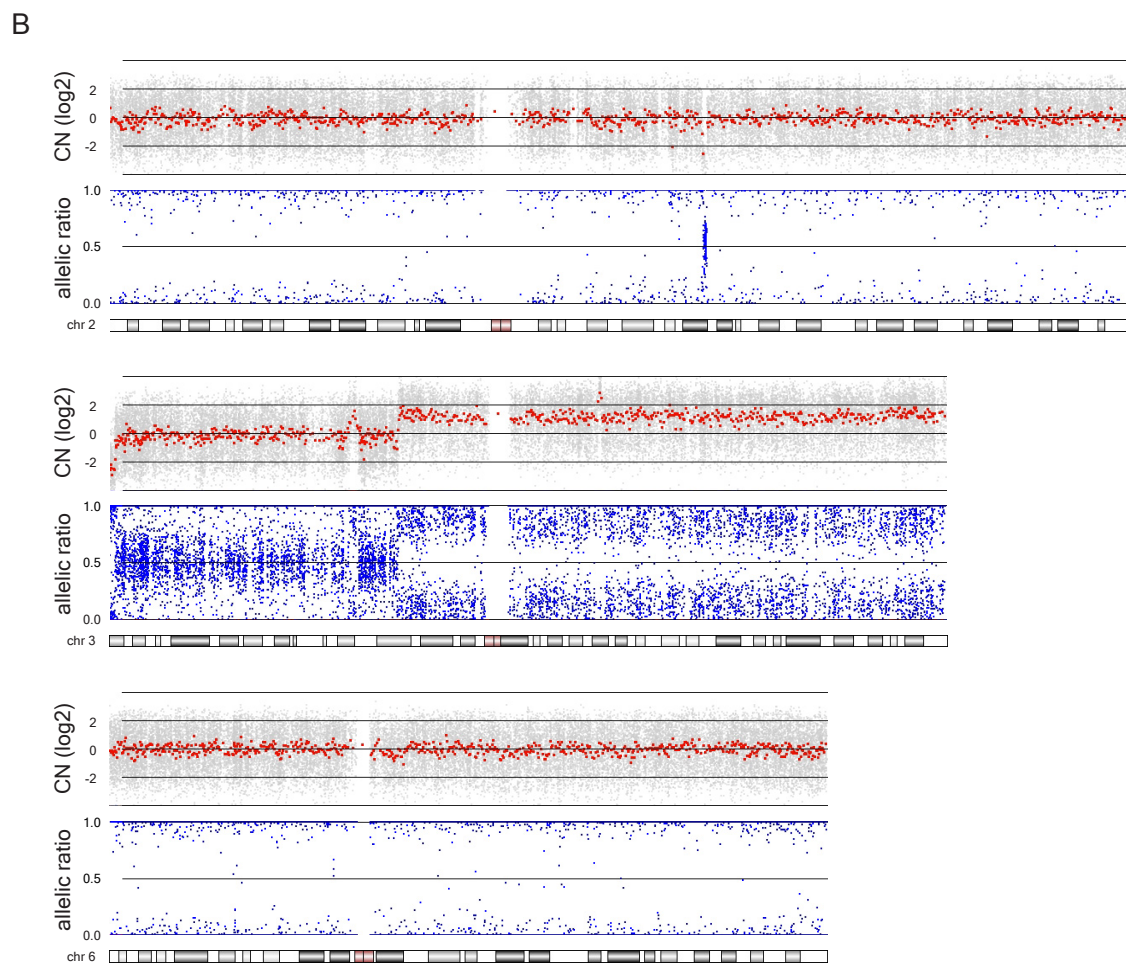

Supplement: Figure S2 — A. DNA copy number variation is shown with a heatmap. The tick marks on the left show boundaries between the two adjacent chromosomes. The color scale indicated at the bottom depicts DNA copy number from 0 to > = 4.0 (blue represents the maximum deletion; tan represents the maximum amplification). B. Genetic alteration at chromosomes 2, 3 and 6 in MCF10CA1a cells. The graph was generated using Partek genomic suite software. DNA copy number (CN) is displayed as log2 ratio against the diploid control (in red). Allelic ratio (displayed in blue) represents the ratio of A-allele CN value divided by (A-allele + B-allele) CN value. Cytogenetic ideograms of chromosomes are displayed on the x-axes. Loss of heterozygosity (LOH) due to either mitotic recombination or chromosomal non-disjunction in chromosomes 2 and 6 are evident from the allele specific analysis. Chromosome 3 displays chromosomal gain (trisomy) in MCF10CA1a cells. Note that most of the normal p-arm on chromosome 3 has log2(CN ratio) value of 0 (diploid) with an allelic ratio of 0.5. C. Intragenic DNA deletion within the LRP1B locus in the MCF10CA1h and MCF10CA1a cell lines. Intragenic deletion in LRP1B is present in the MCF10CA1a and MCF10CA1a cell lines. Note that the transcript is oriented from right to left in this figure. (2.45 MB PDF) [file pone.0009201.s002.pdf]

A

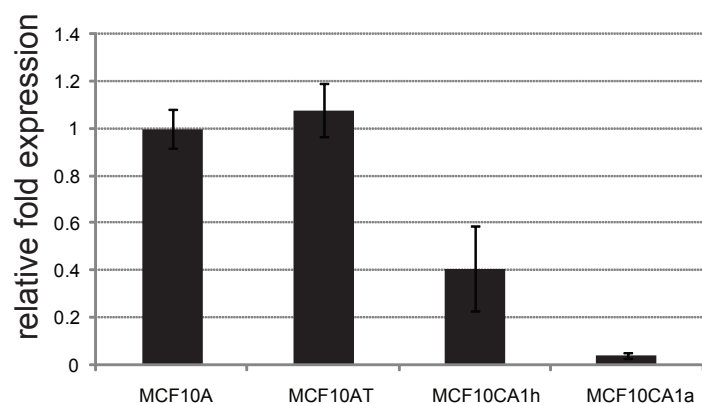

B

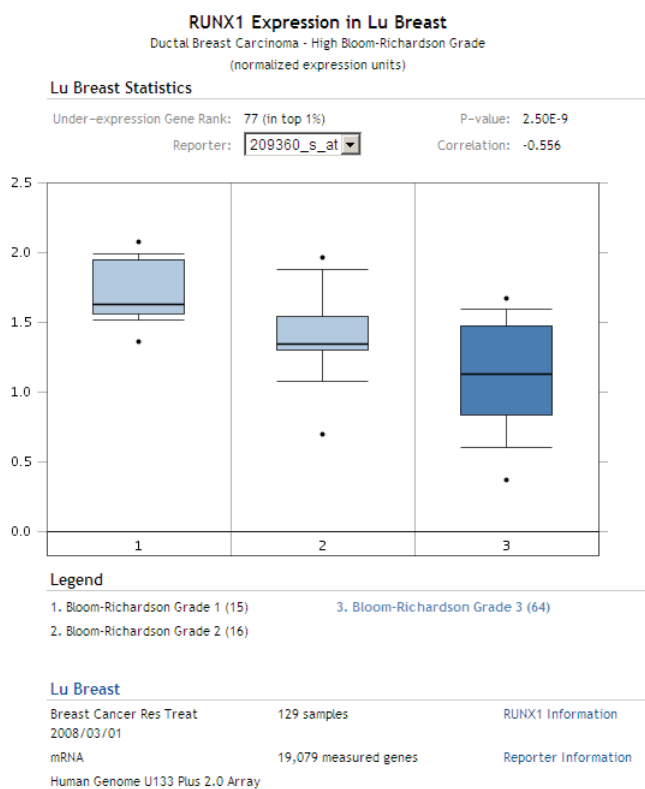

Supplement: Figure S3 — A. Quantitative RT-PCR analysis of the RUNX1 short transcripts. A primer was designed within the 5 prime end of exon 1 in the two short transcripts (NM_001001890 and NM_001122607) that is absent in the long transcript (NM_001754). Quantitative qPCR was carried out using Power SYBR Master Mix (Applied Biosystems) and RUNX1_variant2_forward and RUNX1_variant2_reverse primers at 95°C for 15 sec and 60°C for 1 min for 40 cycles. The reaction products were analyzed with ABI Prism 7900HT sequence detection system (Applied Biosystems). Taqman gene expression analysis against PPIA gene (Applied Biosystems, Hs99999904_m1) was used as a control for normalizing the amount of RNA in the reaction. The short transcripts are expressed in MCF10A, MCF10AT, and MCF10CA1h but absent in MCF10CA1a. Data are shown as mean +/− standard deviation of triplicate measurments. B. RUNX1 gene expression analysis using Oncomine database Breast cancer dataset showed reduced RUNX1 gene expression in high grade tumor. Lu breast dataset (n = 129) on Affymetrix U133 Plus 2.0 array also showed reduced RUNX1 (reporter: 209360_s_at) gene expression in Grade 3 tumor (n = 64) compared to lower grade tumor (n = 15 for Grade 1 and n = 16 for Grade 2). Represented in the box plots are, maximum and minimum values, whiskers at 90th and 10th percentile, boxes at 75th and 25th percentile, and the median. Oncomine (Compendia Bioscience, Ann Arbor, MI) was used for analysis and visualization. (0.80 MB PDF) [file pone.0009201.s003.pdf]
